# Detection and attribution of intra-annual mass component of sea-level variations along the Norwegian coast

**DOI:** 10.1038/s41598-023-40853-8

**Published:** 2023-09-15

**Authors:** Fabio Mangini, Antonio Bonaduce, Léon Chafik, Roshin Raj, Laurent Bertino

**Affiliations:** 1 Nansen Environmental and Remote Sensing Center and Bjerknes Centre for Climate Research, Bergen, Norway; 2 Department of Meteorology and Bolin Centre for Climate Research, Stockholm University, Stockholm, Sweden

**Keywords:** Physical oceanography, Attribution

## Abstract

Reliable sea-level observations in coastal regions are needed to assess the impact of sea level on coastal communities and ecosystems. This paper evaluates the ability of in-situ and remote sensing instruments to monitor and help explain the mass component of sea level along the coast of Norway. The general agreement between three different GRACE/GRACE-FO mascon solutions and a combination of satellite altimetry and hydrography gives us confidence to explore the mass component of sea level in coastal areas on intra-annual timescales. At first, the estimates reveal a large spatial-scale coherence of the sea-level mass component on the shelf, which agrees with Ekman theory. Then, they suggest a link between the mass component of sea level and the along-slope wind stress integrated along the eastern boundary of the North Atlantic, which agrees with the theory of poleward propagating coastal trapped waves. These results highlight the potential of the sea-level mass component from GRACE and GRACE-FO, satellite altimetry and the hydrographic stations over the Norwegian shelf. Moreover, they indicate that GRACE and GRACE-FO can be used to monitor and understand the intra-annual variability of the mass component of sea level in the coastal ocean, especially where in-situ measurements are sparse or absent.

## Introduction

Global mean sea level (GMSL) is one of the key indicators of climate change and provides strong evidence that the Earth’s climate is changing^1^. According to past sea-level reconstructions, GMSL has been rising at a rate between 1.3 and 2.0 mm/year since 1900^2–4^, and has been accelerating since the 1960s^5^. Climate models further suggest that GMSL will likely continue to rise at an accelerated rate in the XXI century^6^, even under very low emission scenarios^1^.

While important to assess the state of the Earth’s climate, GMSL cannot be used to quantify the impact of sea-level change on coastal communities and ecosystems^7^. Such a limitation results from local sea-level changes largely departing from the global average^5,8,9^. Local departures are caused by a variety of processes that affect sea level regionally^10^, have a global, but inhomogeneous impact on sea level^11^, or redistribute water within the ocean without altering the global mean^12^. These processes, which can be grouped into four categories, will be now briefly described (for a thorough description of these processes, the reader is referred to Gregory et al.^13^, Hamlington et al.^14^, and Wahl and Dangendorf^15^). At first, on a regional scale, sea level responds to changes in mean sea-level pressure through the inverse barometer effect. Secondly, sea level is affected by sterodynamic variations of the ocean. Sterodynamic variations are steric in origin and result from changes in ocean currents and in the fluxes of heat and freshwater at the surface and at the lateral boundaries of the ocean. Thirdly, regional sea-level variability is induced by changes in the Earth’s gravity, rotation, and deformation, which occur in response to variations in land-based ice. Finally, sea-level observations, when referred to land (as for the tide gauges), are affected by vertical movements of the Earth’s crust.

Over the years, sea-level changes have been monitored and explained on a local scale^12^. This helped evaluate the quality and the limitations of the present observing system^16^, and constrain the sea-level contributions that are still little known, such as the role of the deep ocean^17^. This also helped understand and quantify the mechanisms behind the observed sea-level variations: a knowledge needed to assess the ability of climate models to reproduce sea-level changes that occurred in the past and might occur in the future^18^, and to filter out the higher frequency sea-level variability, such as the inter-annual and decadal timescales, that prevent accurate estimates of regional sea-level trends^19^.

The present work is a regional sea-level study that explores the contribution of local and remote winds to the mass component of sea level along the Norwegian coast on intra-annual timescales. This paper complements and expands the work by Richter et al.^20^ and Calafat et al.^21^. It studies the contribution of winds to the mass component of sea-level variability in the region using both large-scale atmospheric patterns and the wind stress blowing along the European continental slope. Furthermore, it uses two sets of estimates of the mass component of sea level in the region: one is derived from the difference between the total sea-level signal and its steric component, whereas the other is provided by the Gravity Recovery and Climate Experiment (GRACE) satellite gravimetry mission and its successor, GRACE-FO (for convenience, both GRACE and GRACE-FO are referred to as GRACE in the manuscript).

**Figure 1:**
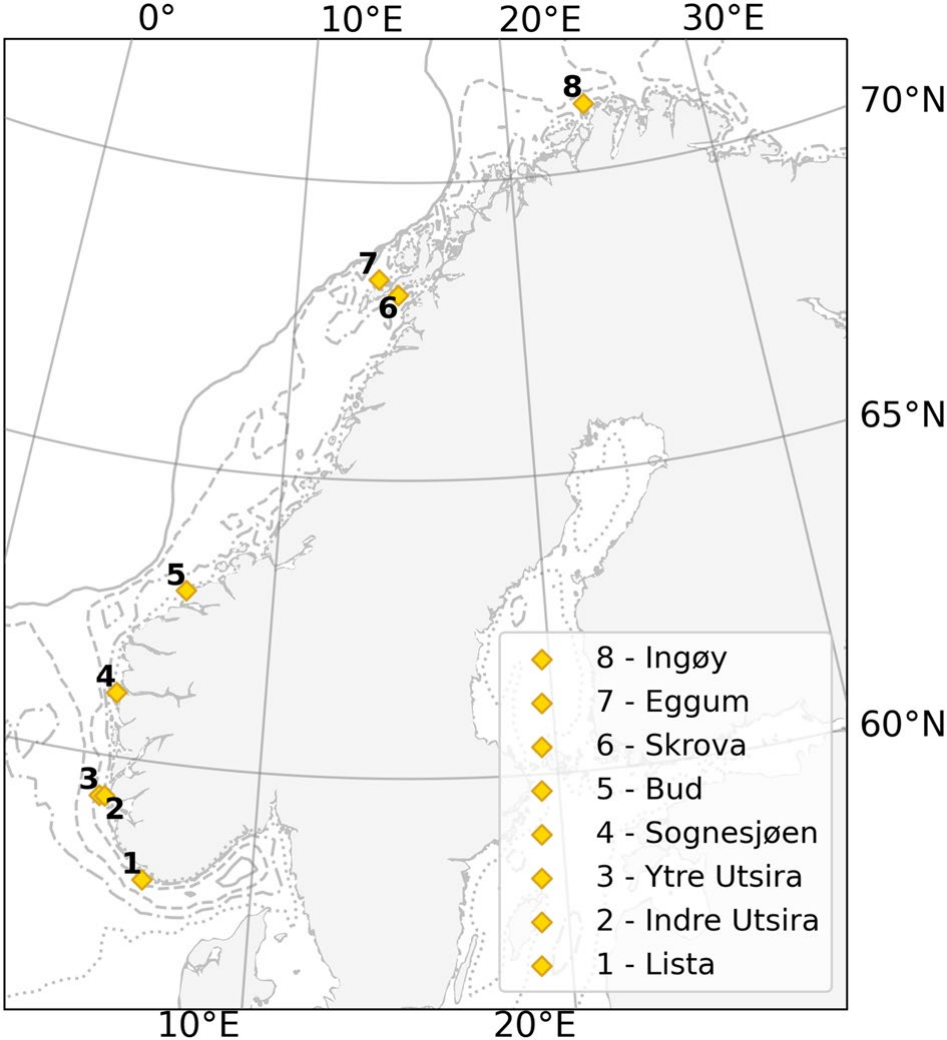
Location of the eight permanent hydrographic stations along the coast of Norway.

This paper employs both GRACE and a combination of satellite altimetry and hydrographic stations to obtain two independent estimates of the mass component of sea level along the coast of Norway. By doing so, it aims to reach more robust conclusions and to assess the quality of the present observing system. This last attempt is important because of the instruments’ limitations. The hydrographic stations do not provide a full coverage of the Norwegian coast and might not give the full picture of temperature and salinity in the region (Fig. 1). Furthermore, their estimates might be influenced by very local processes. For example, the temperature and salinity profiles measured by the station at Sognesjøen (hydrographic station number 4 in Fig. 1) might be strongly affected by the circulation of the Sognefjord.

GRACE has helped monitor and understand the causes of sea-level change over the open ocean^22^, but has historically been considered unreliable in coastal areas^23,24^. The unreliability of GRACE in the coastal zone largely depended on the gravitational field from GRACE being conventionally expressed in terms of spherical harmonics^25^. Because of GRACE’s coarse spatial and temporal resolutions (300 km and 30 days respectively), the spherical harmonic expansion is commonly truncated at degree and order 60 since higher degrees and orders only contain a small fraction of the geophysical signal^26^. The truncation, though, leads to signal leakage, especially in coastal areas because of the different amplitude and variability of the water content over land and the nearby ocean^27^.

To overcome the limitation of the spherical harmonic approach, different mass concentration (mascon) solutions have been devised. The mascon approach involves the pixelization of the Earth’s surface into cells, with each cell characterized by its own uniform mass and its own gravitational signal. The gravitational field of the Earth is then given by the sum of the contribution of each cell. Different mascon solutions may differ by the size and shape of the cells, and by how the GRACE observations are reprocessed to reconstruct the gravitational field associated with each cell^24^.

Because the mascon approach has shown potential in the coastal zone^28,29^, it has been considered in the present paper to study the mass component of sea level along the coast of Norway on intra-annual timescales. More precisely, this paper opts for three different GRACE mascon solutions: one solution is provided by NASA Goddard Space Flight Center (GSFC; Loomis et al.^30^ and Loomis et al.^31^), one by NASA Jet Propulsion Laboratory (JPL; Watkins et al.^24^), and the last one by the Center for Space Research (CSR; Save et al.^32^). The choice of multiple mascon solutions helps determine the robustness of the results and identify features that are present in the GRACE data, but result from their reprocessing and, therefore, have no physical meaning. By doing so, this paper attempts to assess the ability of this recent advance in remote sensing to capture regional sea-level variations.

This paper also provides a physical evaluation of the estimates from GRACE and from the combination of satellite altimetry and the hydrographic stations. Along the coast of Norway, local winds are expected to explain a large fraction of the intra-annual mass component of sea-level variability in the region through Ekman transport^20,21^: local winds blowing along the Northern European continental slope either push water onto the Norwegian shelf or away from it, therefore inducing a mass signal along the coast of Norway. This evaluation is of interest because, to the authors’ knowledge, such a comparison has never been performed on intra-annual timescales.

The paper is organized as follows. At first, it shows that the mass component of sea level explains a large faction of the full sea-level signal along the Norwegian coast. Then, it evaluates the ability of GRACE, satellite altimetry and the Norwegian hydrographic stations to reproduce the mass component of sea-level variability on intra-annual timescales through an inter-comparison between the datasets and through a physical evaluation based on the role played by local winds. It finally quantifies the contribution of local and remote winds to the intra-annual mass component of sea level in the region. It ends with a description of the main conclusions, and of the datasets and methodology used in the study.

## Results

### Importance of the mass component of sea-level variability on intra-annual timescales

**Figure 2:**
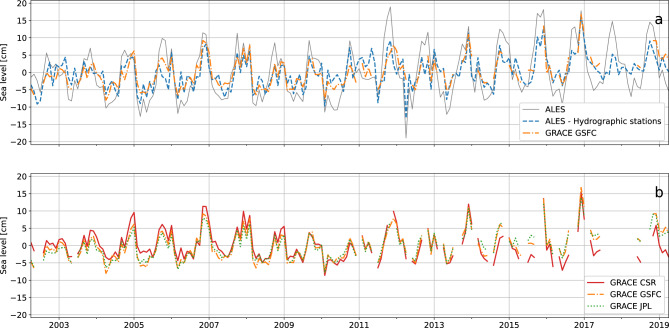
(**a**) Sea level from satellite altimetry (gray, solid line), mass component of sea level from the GSFC mascon solution (orange, dashed-dotted line) and from the combination of satellite altimetry and the hydrographic stations (blue, dashed line) averaged along the Norwegian coast. (**b**) Mass component of sea level from the GSFC mascon solution (orange, dashed-dotted line), the JPL mascon solution (green, dotted line) and the CSR mascon solution (red, solid line).

The mass component of sea level contributes significantly to the total sea-level signal averaged along the coast of Norway (the reader is referred to the subsection “Average along the Norwegian coast” in the Methods to understand how the average along the coast Norway is performed). The contribution of the sea-level mass component appears from simple visual inspection (Fig. 2) as well as from a more quantitative analysis. For example, the ratio between the range of the sea-level mass component from the GSFC mascon solution and the full sea-level signal is approximately 2/3 (25 cm vs 38 cm). Furthermore, the standard deviation of the full sea-level signal decreases by approximately 40% when subtracting the sea-level mass component (7.3 cm vs 4.3 cm).

**Figure 3:**
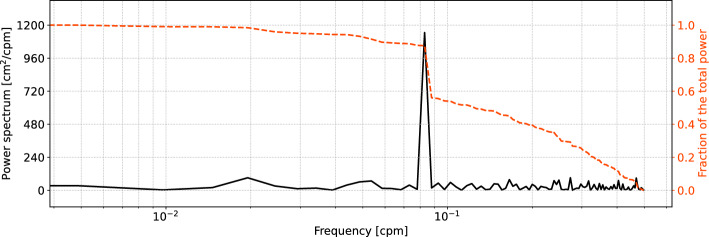
The black, solid line shows the power spectrum of the mass component of sea level averaged along the Norwegian coast that has been estimated from satellite altimetry and the hydrographic stations. The orange, dashed line shows the fraction of power that is contained within the frequency band ranging from 0.49 cpm (cycles per month), the highest frequency, to the frequency indicated on the x-axis.

As previously stated, this paper considers the sea-level mass component on intra-annual timescales (the reader is referred to the last subsection of the Methods for information on the high pass filtering technique). It favours the intra-annual variations because they explain a large fraction of the variance of the mass component of sea-level variability (approximately 56%) averaged along the coast of Norway. This is shown in the power spectrum of the sea-level mass component obtained from the combination of satellite altimetry and the hydrographic stations (also referred to as the “altimetry-based solution” in this manuscript) averaged along the Norwegian coast (Fig. 3). The power spectrum is produced using the altimetry-based solution because, contrary to the GRACE data, it has no missing values. However, the choice of the dataset is expected to only little affect this conclusion because of the good agreement among the four estimates.

While interesting from an oceanographic perspective, this paper will not focus on the inter-annual timescales. An attempt to assess the quality of GRACE on inter-annual timescales has returned a poor agreement with the winds and the altimetry-based estimates. Such a disagreement might result from technical reasons, possibly due to the reprocessing of the GRACE dataset, and not to the inability of GRACE to capture inter-annual sea-level variations. Further analysis is needed, and we plan to address the inter-annual variations of the mass component of sea level in a later study.

### Mass component of sea level: comparison with local winds

This section examines how local winds compare to the mass component of sea-level variability on intra-annual timescales. Such a comparison aims to verify the quality of the altimetry-based solution and the GRACE datasets along the Norwegian coast. At the same time, it aims to quantify the contribution of local winds to the sea-level mass component in the region.

**Figure 4:**
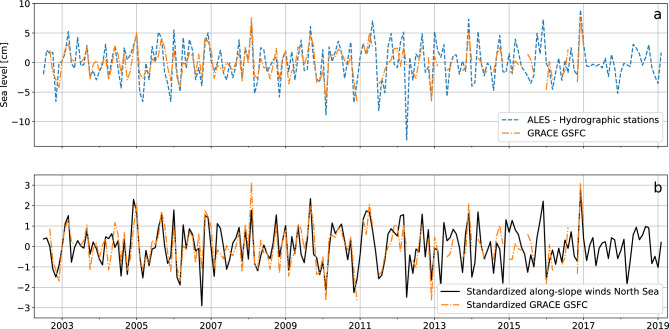
(**a**) Mass component of sea level averaged along the Norwegian coast estimated from satellite altimetry and the hydrographic stations (blue, dashed line) and from the GSFC mascon solution (orange, dashed-dotted line). (**b**) Standardized along-slope component of the wind stress averaged along the North Sea section of the continental slope (black, solid line) compared with the standardized mass component of sea level averaged along the Norwegian coast estimated from the GSFC mascon solution (orange, dashed-dotted line). All the time series has been high pass filtered before plotting to only show the intra-annual variations.

Overall, the intra-annual mass component of sea level from the GSFC’s mascon solution well agrees with the altimetry-based solution (Fig. 4a). The two time series are highly correlated (correlation of 0.70, significant at a 0.05 significance level) and have comparable amplitudes (they mostly oscillate between − 5 and 5 cm). Furthermore, the similarity between GRACE and the altimetry-based solutions seems robust as it is not limited to the product provided by the GSFC, but also holds for the JPL’s and CSR’s mascon solutions (Fig. S1 in the Supplementary materials).

Figure 4a,b, and Supplementary Fig. S1 show that both the mass component of sea level from GRACE and the satellite-based solution agree as expected with the along-slope wind stress averaged along the North Sea section of the continental slope on intra-annual timescales. A still good, but slightly worse agreement is also found with the along-slope wind stress averaged along the Norwegian section of the continental slope (not shown). Such a good agreement confirms the ability of GRACE, satellite altimetry and the hydrographic stations to reproduce the mass component of sea level along the Norwegian coast. At the same time, the altimetry-based solution and the estimates from GRACE indicate that local winds explain between approximately 49% and 63% of the variance of the mass component of sea level in the region.

**Figure 5:**
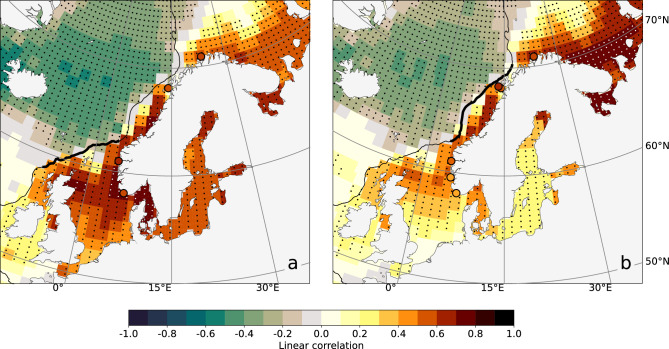
(**a**) Linear correlation coefficient between the along-slope component of the wind stress averaged along the North Sea section of the northern European continental slope (the black, thick line) and the mass component of sea level from GSFC’s mascon solution (shading) and from the combination of ALES and the hydrographic stations (circles) on intra-annual timescales. (**b**) Same as (**a**), but the linear correlation is performed with the wind stress averaged along the Norwegian section of the northern European continental slope. The black and white dots identify the regions where the linear correlation is significant at a 0.05 significance level. Furthermore, only the hydrographic stations for which the linear correlation coefficients are significantly different from zero at a 0.05 significance level are shown. The black, thin line shows the location of the continental slope, depicted by the 500 m isobath.

While less reliable for the reasons described in the “Methods” section (see the “Average along the Norwegian coast” subsection), the paper also presents a point-wise analysis of the intra-annual mass component of sea level in northern Europe. The paper assesses the contribution of wind stress to the mass component of sea level along the Norwegian coast on intra-annual timescales through linear correlation. At first, the intra-annual along-slope component of the wind stress is averaged, respectively, over the North Sea and the Norwegian sectors of the northern European continental slope (as a convention, the along-slope winds are set to be positive when blowing along the north-eastward direction). Then, the result is correlated with the intra-annual mass component of sea level from GRACE and from the combination of ALES and the hydrographic profiles. Because the correlation maps are mostly independent on the choice of the GRACE dataset, the remaining part of the section only describes the results for the GSFC mascon solution (Fig. 5), leaving the results for the JPL and the CSR mascon solutions to the Supplementary material (Figs. S2 and S3).

GRACE and the combination of satellite altimetry and hydrographic stations clearly show the role of local winds in driving the mass component of sea level over the northern European continental shelf on intra-annual timescales. In agreement with Ekman theory, both Fig. 5a,b show negative values over the Nordic Seas and positive values over the northern European continental shelf. The estimates of the mass component of sea level from satellite altimetry and the hydrographic stations return consistent results. They positively correlate with the local winds and differ on average by less than 0.15 percentage points from the linear correlation coefficients returned by GRACE in proximity of the hydrographic stations.

Both sets of estimates also help identify variations of the sea-level mass component within the continental shelf. As expected from intuition, the along-slope winds blowing along the North Sea section of the continental slope mostly relate to variations in the mass component of sea level in the North Sea and western Norway. Instead, the winds blowing along the Norwegian section of the slope show higher correlations with the mass component of sea level in northern Norway and the Barents Sea. GRACE also shows a spatial variation in the North Sea, with the along-slope wind stress being more strongly correlated to the mass component of sea level in the northern North Sea than in the southern North Sea. This is in line with the existing literature which relate sea-level variations in the southern North Sea to the winds blowing over the entire North Sea domain, and not only along the continental slope^33–37^.

In summary, the correlation maps show an overall good agreement between GRACE and the combination of satellite altimetry and hydrographic stations. These results are promising as they suggest that both sets of estimates capture the contribution of local winds along the coast of Norway.

### Mass component of sea level: a synoptic view

This section favours a synoptic view and identifies the large-scale atmospheric conditions associated with the sea-level variations previously described. This approach helps introduce a simple atmospheric index to reproduce the mass component of sea level along the Norwegian coast on intra-annual timescales. At the same time, it allows a comparison with the North Atlantic Oscillation (NAO), the main mode of atmospheric variability in the North Atlantic^38^.

**Figure 6:**
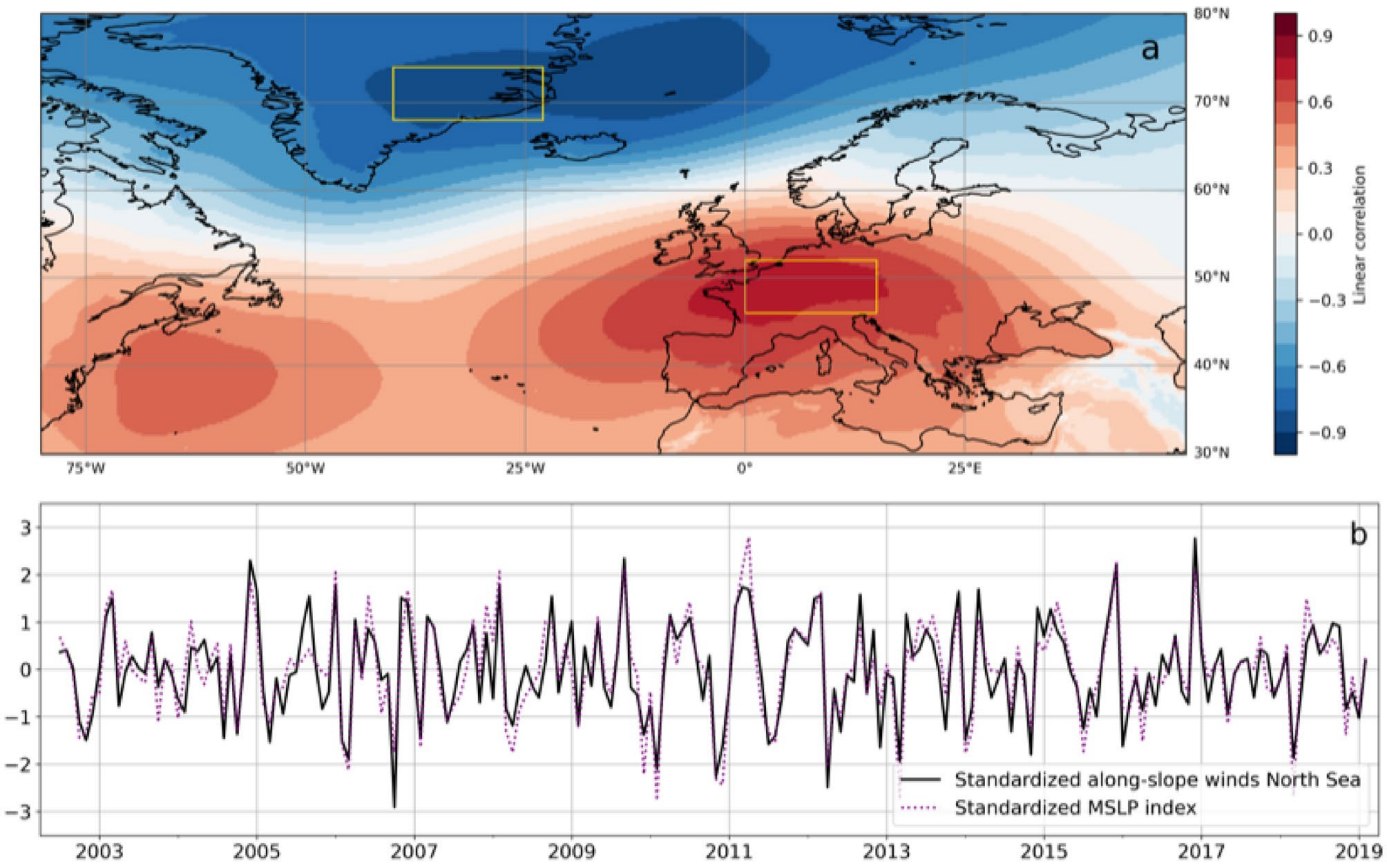
(**a**) Linear correlation coefficient between the gridded intra-annual monthly MSLP and the intra-annual along-slope component of the along-slope wind stress averaged over the North Sea section of the northern European continental slope. (**b**) Standardized intra-annual along-slope component of the along-slope wind stress averaged along the North Sea section of the continental slope (black, solid line) compared with the standardized MSLP index (purple, dotted line).

The large-scale atmospheric pattern is identified through simple linear correlation. More specifically, the intra-annual monthly MSLP is correlated with the along-slope component of the intra-annual wind stress averaged along the North Sea section of the continental slope (Fig. 6a). This procedure returns a large-scale pattern with two main centres of actions: one is located at circa 70° N over eastern Greenland and over the Iceland and Greenland Seas, whereas the other over northern France and Germany. While statistical in origin, the relationship between the large-scale atmospheric pattern and the along-slope wind stress makes physical sense as the maximum MSLP gradient is located along the North Sea section of the northern European continental slope.

As previously stated, the large-scale pattern in Fig. 6a helps create a simple index to reconstruct the mass component of sea level along the Norwegian coast. The MSLP index is built as the difference between the monthly MSLP averaged over the southern and the northern centres of action (yellow polygons in Fig. 6a). It is found that the MSLP index well agrees with the along-slope wind stress through visual inspection (Fig. 6b) and through their high linear correlation (r = 0.89, significantly different from zero at 0.05 significance level).

The large-scale pattern is also found to agree relatively well with the NAO. A comparison with the empirical orthogonal functions of the intra-annual MSLP over the North Atlantic and northern Europe (30–80° N, 80° W–50° E) shows that the large-scale pattern in Fig. 6a resembles the leading mode of atmospheric variability over the North Atlantic (Fig. S4 in the Supplementary material). Indeed, the NAO has one centre of action located over the eastern side of central Greenland and the other over the North Atlantic Ocean west of northern Spain. Furthermore, the linear correlation coefficient between the MSLP index and the NAO index is 0.83, significantly different from zero at 0.05 significance level. Despite this similarity, though, the principal component of the NAO explains a lower fraction of the mass component of sea level along the Norwegian coast on intra-annual timescales when compared with the MSLP index. Depending on the solution of the sea-level mass component, the NAO explains between 24 and 52% of the variance (39% of the variance on average), whereas the MSLP index explains between 43 and 57% of the variance (50% of the variance on average).

### A link to the eastern boundary of the North Atlantic

The previous sections quantified the contribution of local winds to the intra-annual mass component of sea-level variability along the Norwegian coast. The present one addresses the contribution of remote winds.

Winds blowing along the eastern boundary of the North Atlantic are known to contribute to sea-level variations along the western and northern European coast on inter-annual or longer timescales^21,39–41^. The along-slope winds set up an Ekman transport which, through upwelling and downwelling, affects the depth of the thermocline and, in turn, modify sea level^42^. However, the perturbation of the thermocline and sea level are not constrained to where the winds blow, but they propagate over continental shelves as coastal trapped waves^43^. Due to the Earth’s rotation, coastal trapped waves propagate with the coast on their right in the northern hemisphere and on their left in the southern hemisphere.

To assess the contribution of remote winds to the sea-level mass component along the Norwegian coast, the along-slope wind stress is integrated along the European continental slope from 35° N to 54° N circa (approximately, the continental shelf break of Spain, France, and the UK). The northern edge at 54° N was chosen to ensure that the integrated winds are uncorrelated with the winds averaged along the North Sea section of the continental slope. The southern edge at 35° N was selected to exclude the contribution of coastal trapped waves that form along the north-western coast of Africa. Indeed, these are expected to dissipate before reaching Norway since coastal trapped waves typically dissipate over a few-day period^41^ and have a propagation speed that ranges between 1 and 3 m/s^44^. However, a sensitivity test indicates that the choice of a lower latitude for the southern edge only plays a secondary role as it only little affects the results (not shown).

**Figure 7:**
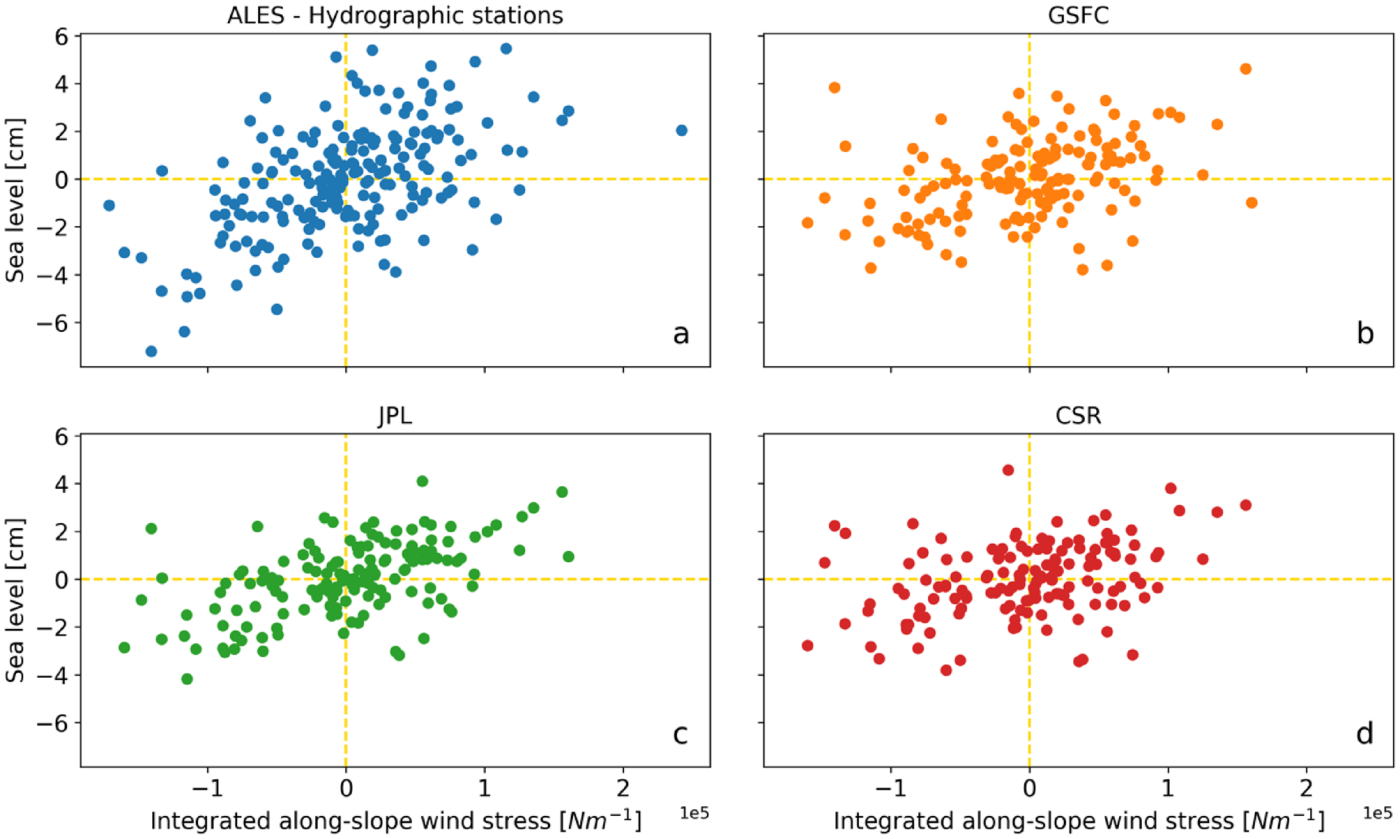
(**a**) Intra-annual mass component of sea level estimated using a combination of satellite altimetry and hydrography from which the contribution of local winds has been removed plotted against the intra-annual along-slope wind stress integrated along the western European continental slope. (**b**), (**c**) and (**d**) are like (**a**), but show the results for the GSFC’s, the JPL’s, and the CSR’s mascon solutions respectively.

The results indicate that remote winds contribute to intra-annual sea-level variations along the Norwegian shelf. This is deduced from simple visual inspection (Fig. 7) as well as from a more quantitative analysis. To quantify the contribution of remote winds, the integrated intra-annual along-slope wind stress is linearly correlated with the intra-annual mass component of sea level from GRACE and the altimetry-based solutions. It is found that the linear correlation coefficients range from a minimum of 0.26, for the CSR’s GRACE mascon solution, to a maximum of 0.42, for the JPL’s mascon solution. However, despite the positive correlations, only the results for the JPL’s product and the altimetry-based solution are significantly different from zero at a 0.05 significance level.

The impact of remote winds becomes clearer when the procedure described in the previous paragraph is repeated, this time removing the contribution of local winds from the sea-level mass component averaged along the Norwegian coast. This procedure returns correlation coefficients that are both higher than the ones presented in the previous paragraph, as they range from 0.35 to 0.54, and are significantly different from zero at a 0.05 significance level.

The role of remote winds also becomes more apparent through the construction of a multiple linear regression model which reproduces the mass component of sea level using as predictors both the along-slope wind stress averaged along the North Sea section of the continental break and the along-slope wind stress integrated along the eastern margin of the North Atlantic. The variance of the intra-annual mass component of sea-level variability explained by the model ranges between 61 and 68%, with an average increase of 10 percentage points over the linear regression model presented in the section “Mass component of sea level: comparison with local winds”.

## Discussion and conclusions

This paper examines the mass component of sea level along the Norwegian coast on intra-annual timescales. At first, it assesses the ability of GRACE, satellite altimetry, and the Norwegian hydrographic stations to estimate changes in ocean mass over the Norwegian shelf. Then, it explores the relationship between these estimates and atmospheric reanalysis data to explain part of the intra-annual mass component of sea-level variability in the region.

As a main result, the paper finds that the GRACE mascon solutions provided by the GSFC, JPL and CSR, as well as the combination of satellite altimetry and the hydrographic stations, return reliable estimates of the mass component of sea level averaged along the Norwegian coast on intra-annual timescales. The paper bases this conclusion on the high degree of similarity between the two sets of independent estimates, the ones from GRACE and the one from the altimetry-based solution. The paper corroborates this finding with a physically based evaluation of the two sets of estimates. More precisely, it shows that they well agree with the along-slope wind stress averaged, respectively, along the North Sea section and the Norwegian section of the continental slope. This result is of interest for a couple of reasons. At first, it is line with the existing literature^28,29^ which indicates the potential of the GRACE mascon solution in coastal areas, where the GRACE and GRACE-FO satellite missions have historically been considered little reliable^25^. Secondly, it highlights the ability of the Norwegian hydrographic stations to capture temperature and salinity variations that are representative for the entire Norwegian shelf even though, a-priori, they might be affected by very local processes.

The paper also suggests that GRACE and the combination of satellite altimetry and the hydrographic stations capture the spatial variability of the sea-level mass component over the northern European continental shelf. The paper reaches this conclusion through a point-wise analysis of GRACE and the altimetry-based solution. The correlation maps with the along-slope wind stress averaged along the North Sea and the Norwegian sections of the continental slope, respectively, show a latitudinal dependence of the mass component of sea level on the along-slope winds: the along-slope winds blowing along the North Sea section of the continental slope mostly affect sea-level variations in the North Sea and the southern portion of the Norwegian shelf, whereas those blowing along the Norwegian section of the slope affect sea-level variations along the northern Norwegian coast and the Barents Sea. Furthermore, the correlation maps show that all GRACE mascon solutions detect the presence of the northern European continental slope despite GRACE’s and GRACE-FO’s coarse spatial resolutions.

Together with the impact of longshore winds, the paper also investigates the contribution of large-scale atmospheric forcings on the intra-annual mass component of sea-level variations in the region. The paper finds that the along-slope wind stress averaged along the continental break of the North Sea projects onto one single large-scale atmospheric pattern whose centres of action are located over the central areas of western Greenland, and over France and Germany. This relationship is then used to build a simple MSLP index to reproduce the intra-annual variability of the along-slope wind stress and, consequently, of the mass component of sea level in the region. The MSLP index is of interest because it is easier to build when compared with the along-slope wind stress averaged along the continental slope of the North Sea. Furthermore, depending on the presence of barometers in the locations of the two centres of action during the nineteenth and twentieth century, the MSLP index opens up to the possibility of reconstructing past variations of the mass component of sea level along the Norwegian coast.

The large-scale atmospheric pattern identified in the paper also allows a comparison with the NAO. The paper shows the similarity between the two large-scale atmospheric patterns which, in turn, explains why Richter et al.^20^ could use the NAO to study the contribution of local winds to the mass component of sea-level variability in the region. Furthermore, the paper highlights the importance for climate models to reproduce the NAO to properly simulate the contribution of winds to the mass component of sea level in the area.

Previous work attributed inter-annual and decadal sea-level variations in northern Europe to the winds blowing along the eastern boundary of the North Atlantic^19,21,45^. This paper finds that remote winds also contribute to the Norwegian mass component of sea level on intra-annual timescales, even though to a lesser extent when compared with local winds. This result supports the hypothesis that coastal trapped waves drive the sea-level variability along the coast of Norway on intra-annual timescales. Moreover, this result hints at the structure of the coastal trapped waves that are responsible for these variations: the amplitude of coastal trapped waves must be different from zero in the coastal areas of Norway for it to be measured by the hydrographic stations, which are located within 3 and 15 km from the coast of Norway^46^. This last result agrees with Hughes et al.^41^ who suggests a role for coastal trapped waves of mode-0 and mode-1 in driving sea-level variations in coastal areas. Indeed, mode-0 and mode-1 are the most naturally generated by large-scale forcing and they usually have the highest elevation at the coast.

Overall, the results found over the Norwegian shelf give confidence that both GRACE/GRACE-FO and the combination of satellite altimetry and hydrographic stations can be used to analyse sea-level variations in other areas of the coastal ocean. Furthermore, the results could be the base of future work with GRACE data. For example, a future study could determine whether the ability of GRACE and GRACE-FO to measure the mass component of sea level depends on the shape of continental shelves. Another could follow and extend the example in Landerer et al.^47^ and use GRACE data to estimate ocean transports in coastal areas.

## Data

### GRACE: GSFC’s mascon solution

This paper uses GRACE gridded data from NASA Goddard Space Flight Center. The data are provided as equivalent height of sea level (assuming water has a density of 1000 kg/m^3^), with spatial resolution of 0.5° × 0.5° and a temporal resolution of one month. The dataset covers the period from April 2002 to December 2021. However, only the period from April 2002 to April 2019 is selected for the GSFC’s GRACE dataset to overlap in time with the satellite altimetry dataset (see later in the section).

Several corrections have been applied to the GRACE dataset. The dataset is corrected for the geocenter motion (degree 1)^48^, oblateness (C_20_) and C_30_ from TN-14^49^, and the glacial isostatic adjustment (GIA) using the ICE-6G_D model^50^. Furthermore, the mass of the atmosphere over the ocean has been removed at each grid point of the GRACE dataset to retrieve the mass component of sea level from the ocean bottom pressure (under the assumption that the ocean is incompressible).

For more information on the dataset, the reader is referred to Loomis et al.^30^ and Loomis et al.^31^.

### GRACE: JPL’s mascon solution

This paper uses GRACE gridded data from NASA Jet Propulsion Laboratory. The dataset is provided as equivalent height of sea water, under the assumption that water density is 1000 kg/m^3^. The data have a native resolution of 3° × 3° (this is the original mascons size), however they are provided on a regular grid with spatial resolution of 0.5° × 0.5°. The coastline resolution improvement (CRI) filter is applied to the data. This is a post-processing step needed to separate the land and the ocean contribution to the Earth’s gravity field in the mascons that contain both land and ocean. The dataset covers the period from April 2002 to February 2022. However, only the period from April 2002 to April 2019 is selected for the JPL’s GRACE dataset to overlap in time with the satellite altimetry dataset (see later in the section).

Several corrections have been applied to the dataset. The C_20_ coefficients have been substituted with satellite laser ranging^51^, and the degree-1 coefficient has been estimated by applying the method in Swenson et al.^52^. The data has also been corrected for the GIA with the ICE-6G_D model^50^. Moreover, as for the GSFC’s mascon product, the surface atmospheric pressure averaged over the global ocean has been subtracted from the ocean bottom pressure provided by GRACE at each grid point to obtain an estimate of the mass component of sea level.

For more information on the dataset, the reader is referred to Watkins et al.^24^.

### GRACE: Center for Space Research (CSR)

This paper uses GRACE gridded data from the Center for Space Research. The dataset is provided as equivalent height of sea water, under the assumption that water density is 1025 kg/m^3^. The data have a spatial resolution of 0.25° × 0.25°, despite their native resolution being 1° × 1°, and a temporal resolution of one month circa. The dataset covers the period from April 2002 to May 2022. However, as for the GSFC’s and the JPL’s mascon solution, only the period up to April 2019 is selected for the CSR’s GRACE dataset to overlap in time with the satellite altimetry dataset (see later in the section).

Several corrections have been applied to the CSR’s mascon solution. For consistency with the other GRACE and GRACE-FO solutions, the C_20_ coefficients have been replaced with the C_20_ solutions from Loomis et al.^53^. Furthermore, the C_30_ coefficients have been replaced with the C_30_ solutions from Loomis et al.^53^ for the GRACE-FO solutions. Data have also been corrected for the degree-1 gravity coefficients following Sun et al.^48^ and for GIA with the ICE-6G_D model^50^. As for the GSFC’s and the JPL’s mascon product, the surface atmospheric pressure averaged over the global ocean has been removed at each grid point for the data to return the sea-level mass component instead of ocean bottom pressure.

### Hydrographic stations

The Institute for Marine Research (IMR) maintains eight permanent hydrographic stations in the shallow waters along the Norwegian coast (Fig. 1). Most of them were installed in the 1930s and the 1940s to monitor temperature and salinity variations along the coast of Norway due to their relevance for fisheries. Temperature and salinity data are collected by fishermen and local observers trained by IMR approximately once or twice a month^20^. Data are publicly available at http://www.imr.no/forskning/forskningsdata/stasjoner/index.html.

Even though the hydrographic stations cover a significant part of the XX century, this paper only considers the temperature and salinity data over the period between April 2002 and April 2019 (205 months) for them to overlap in time with the satellite altimetry dataset. Over this period, the observations of the hydrographic stations have gaps. These range from a minimum of 3 at Lista, to a maximum of 81 at Bud, but the observations from the hydrographic stations were all deemed usable.

In this project, temperature and salinity profiles at each hydrographic station are used to compute the corresponding monthly mean steric component of sea-level variability. The steric component of sea level is then subtracted from the SLA measured by satellite altimetry to compute the mass component of sea level along the Norwegian coast. These estimates are subsequently compared with the estimates from GRACE.

### ALES-retracked satellite altimetry dataset

To estimate the sea-level variability along the coast of Norway, this paper uses the along-track satellite altimetry dataset reprocessed with the Adaptive Leading Edge Subwaveform (ALES) retracker^54^. This paper opts for the ALES-reprocessed instead of the conventional satellite altimetry dataset because it is more suitable in coastal areas: the ALES retracker only selects the portion of the returned echo that is not perturbed by the presence of land and fits the corresponding waveform with the open ocean Brown functional form. The ALES retracker can retrieve sea-level observations up to 3 km from the coast with an accuracy comparable with that of the open ocean, in regions where conventional altimetry dataset has historically been flagged as not reliable. Previous studies have shown that the ALES satellite altimetry dataset well captures the sea-level variability along a significant fraction of the coastal ocean^54–57^. A more recent study^58^ has evaluated the dataset against the Norwegian set of tide gauges and has found good agreement in the region.

The ALES-retracked satellite altimetry dataset is publicly available at https://openadb.dgfi.tum.de/en/products/adaptive-leading-edge-subwaveform-retracker/. It includes global sea-level observations at a 1 Hz posting rate from the following satellite missions: Envisat (version 3), Jason-1, Jason-1 extended mission, Jason-1 geodetic mission, Jason-2, Jason-2 extended mission, Jason-3, SARAL, SARAL drifting phase. The data have been inter-calibrated to avoid inter-mission biases. They cover the period between January 2002 and April 2019, with the exception of a gap northward of 66° N from November 2010 to March 2013, between the end of the Envisat mission and the beginning of the SARAL mission.

Following the recommendations from the providers, data were discarded if located within 3 km from the coast, if the significant wave height (SWH) exceeded 11 m, and if the standard deviation was larger than 0.2 m. SLA observations were also excluded if they exceeded 1.5 m, instead of 2.5 m as recommended, because Mangini et al.^58^ found that a more stringent condition returns results that agree better with the tide gauges.

The ALES-reprocessed satellite altimetry dataset has been corrected for the dynamic atmospheric correction (DAC) and tides. The DAC correction removes the contribution of both winds and pressure to the sea-level variability at timescales less than 20 days because this signal would otherwise be aliased into the sea-level time series due to the coarse temporal resolution of satellite altimetry. The DAC correction also removes the effect of atmospheric pressure at longer timescales (> 20 days). Tides have been removed with the EOT11a tidal model.

### Wind stress and mean sea level pressure

In this paper, the analysis is partly based on the mean sea level pressure (MSLP) and the zonal and meridional instantaneous turbulent surface stress from the ERA5 reanalysis dataset^59^ of the European Centre for Medium-Range Weather Forecasts (ECMWF). The quality-checked data cover the entire globe, with a spatial resolution of 0.25° × 0.25°, and span the period from 1959 to present, with a temporal resolution of 1 h. For convenience, the instantaneous turbulent surface stress is referred to as surface wind stress in this manuscript.

## Methods

### Mass component sea level from altimetry and hydrographic stations

To estimate the mass component of sea level at each hydrographic station along the coast of Norway, the ALES-reprocessed satellite altimetry dataset is used in combination with the Norwegian set of hydrographic stations. The mass component of sea level is given by the difference between the sea-level anomaly and the steric component of sea level. Therefore, satellite altimetry is employed to reconstruct the monthly mean sea-level variability at each hydrographic station, and the temperature and salinity profiles at each hydrographic station are used to estimate the corresponding monthly averaged steric component of sea level.

To build a monthly mean sea-level time series at each hydrographic station, this paper follows the same procedure as in Mangini et al.^58^. The results of that study indicate that this procedure returns sea-level time series representative of the location occupied by each hydrographic station if the satellite altimetry observations are averaged over each month and within a radius of 200 km from the hydrographic station and a distance of 20 km from the coast.

Temperature and salinity profiles at each hydrographic station are used to estimate the corresponding monthly mean steric component of sea level. More precisely, the following formula is used:

$$\eta = \mathop \smallint \limits_{{ - {\text{H}}}}^{0} \frac{{\rho_{0} - \rho }}{{\rho_{0} }}{\text{dz,}}$$

where the integration is performed over the entire water column, $\rho$ is the density at each depth level, and ${\rho}_{0}$ is a reference density, calculated for T = 0 °C and S = 35 psu.

### Along-slope wind stress

The paper partly bases its results on the along-slope component of wind stress along the eastern margin of the North Atlantic Ocean. The along-slope component of the wind stress is computed by projecting the monthly-averaged detrended and deseasoned surface wind stress onto the 500 m isobath, which is used as a proxy for the continental slope of Africa and Europe.

### Harmonic analysis

To remove the linear trend and the seasonal cycle from the time series analysed in this study, the Levenberg–Marquardt algorithm is used to fit the following function to each time series:

$$\mathrm{z}\left(\mathrm{t}\right)=\mathrm{ a }+\mathrm{ bt }+\mathrm{ c\,sin}\left(\frac{2\pi }{12}\mathrm{t}+\mathrm{d}\right)+\mathrm{ e\,sin}\left(\frac{4\pi }{12}\mathrm{t}+\mathrm{f}\right),$$

where *a* is the offset, *b* the linear trend, *c* and *d* the amplitude and the phase of the annual cycle, *e* and *f* the amplitude and the phase of the semi-annual cycle.

### Average along the Norwegian coast

In this paper, the mass component of sea level is averaged along the Norwegian coast to provide a more reliable comparison between GRACE and the altimetry-based solution.

The comparison becomes more reliable for two main reasons. At first, the average along the Norwegian coast removes random errors that result from the imperfect co-location of the satellite altimetry tracks and the hydrographic stations. Secondly, a point-wise comparison between the two sets of estimates ignores their different spatial resolutions. The hydrographic stations might be representative for an area a few square kilometres wide because their measurements can be affected by local processes. Instead, GRACE has an effective spatial resolution of approximately 300 km even though it is provided on a 0.5° × 0.5° and on a 0.25° × 0.25° regular grid (depending on the GRACE mascon solution). So, the mass component of sea level at each grid point might not have a clear physical meaning, but it might partly result from spatial interpolation.

With average along the Norwegian coast, the manuscript refers to the average of the altimetry-based solution and the GRACE datasets over the hydrographic stations. However, the average of the detrended and deseasoned mass component of sea level is not simply computed over all the available hydrographic stations. Instead, the twin stations Indre Utsira-Ytre Utsira and Eggum–Skrova are first averaged with each other for them to count as one in the final calculation of the mean.While averaging over the hydrographic stations is straightforward for the altimetry-based solution, it is less so for the GRACE datasets as they are provided on regular grids. For the reprocessing of the GRACE data to be consistent with that of the altimetry-based solution, we first select the grid points that are in proximity of each hydrographic station. Then, we follow the procedure that was described in the previous paragraph. As a sensitivity test, the GRACE data were also averaged over the Norwegian continental shelf or, in other words, over the region shallower than 500 m and delimited by the 57° N and 72° N parallels and by the 4° E and 21° E meridians. However, this alternative approach returned comparable results (not shown).

### High pass filtering technique

A 12-month running mean is used to extract the intra-annual variability from the time series considered in the manuscript. However, the high pass filtering technique is applied with a caveat. The 12-month moving average is undefined if there is at least one gap within the twelve-month window. However, such a strong restriction would limit the analysis to relatively short periods because it increases the number of missing values in a time series if the time series contains gaps. For example, this restriction would constrain the analysis to the period between 2004 and mid-2010 since the GRACE datasets have several missing values. Therefore, for the analysis to span a longer period, this restriction is partly relaxed by allowing the running mean to return a real number if the twelve-month window contains three gaps or less.

### Supplementary Information

Supplementary Figures.

## Data Availability

The ALES-retracked satellite altimetry dataset was produced by DGFI-TUM and distributed via OpenADB (https://openadb.dgfi.tum.de; last access: 22 July 2020). The hydrographic station datasets, obtained from the Institute of Marine Research in Bergen, are updated and available at https://www.imr.no/forskning/forskningsdata/stasjoner/index.html (last access: 11 November 2020). The GRACE mascon solution produced by GSFC was provided by Dr. Bryant D. Loomis and is available on request. The GRACE mascon solution produced by JPL was downloaded from https://podaac.jpl.nasa.gov/dataset/TELLUS_GRAC-GRFO_MASCON_CRI_GRID_RL06_V2 (last access: 11/05/2022). The GRACE mascon solution produced by CSR was downloaded from https://www2.csr.utexas.edu/grace/RL06_mascons.html (last access: 09/09/2022). The ECMWF’s ERA5 dataset was downloaded through the CDS API (https://confluence.ecmwf.int/display/CKB/How+to+download+ERA5#HowtodownloadERA5-First:InstallCDSAPIonyourmachine).
