# Supplementary material for: Detection and attribution of intra-annual mass component of sea-level variations along the Norwegian coast
Source: Sci Rep. 2023 Sep 15;13:15334. doi: 10.1038/s41598-023-40853-8 (PMC10504360; doi:10.1038/s41598-023-40853-8)
Supplement: Supplementary file 1 — Supplementary Figures. [file 41598_2023_40853_MOESM1_ESM.pdf]

Detection and attribution of intra-annual mass component of sea-level variations along the Norwegian coast

Fabio Mangini[1], Antonio Bonaduce[1], Léon Chafik[2], Roshin Raj[1], Laurent Bertino[1]

[1]Nansen Environmental and Remote Sensing Center and Bjerknes Centre for Climate Research, Bergen, Norway

[2]Department of Meteorology and Bolin Centre for Climate Research, Stockholm University, Stockholm, Sweden

Correspondence to: Fabio Mangini (fabio.mangini@nersc.no)

Supplementary material

A - Mass component of sea level: Norwegian average

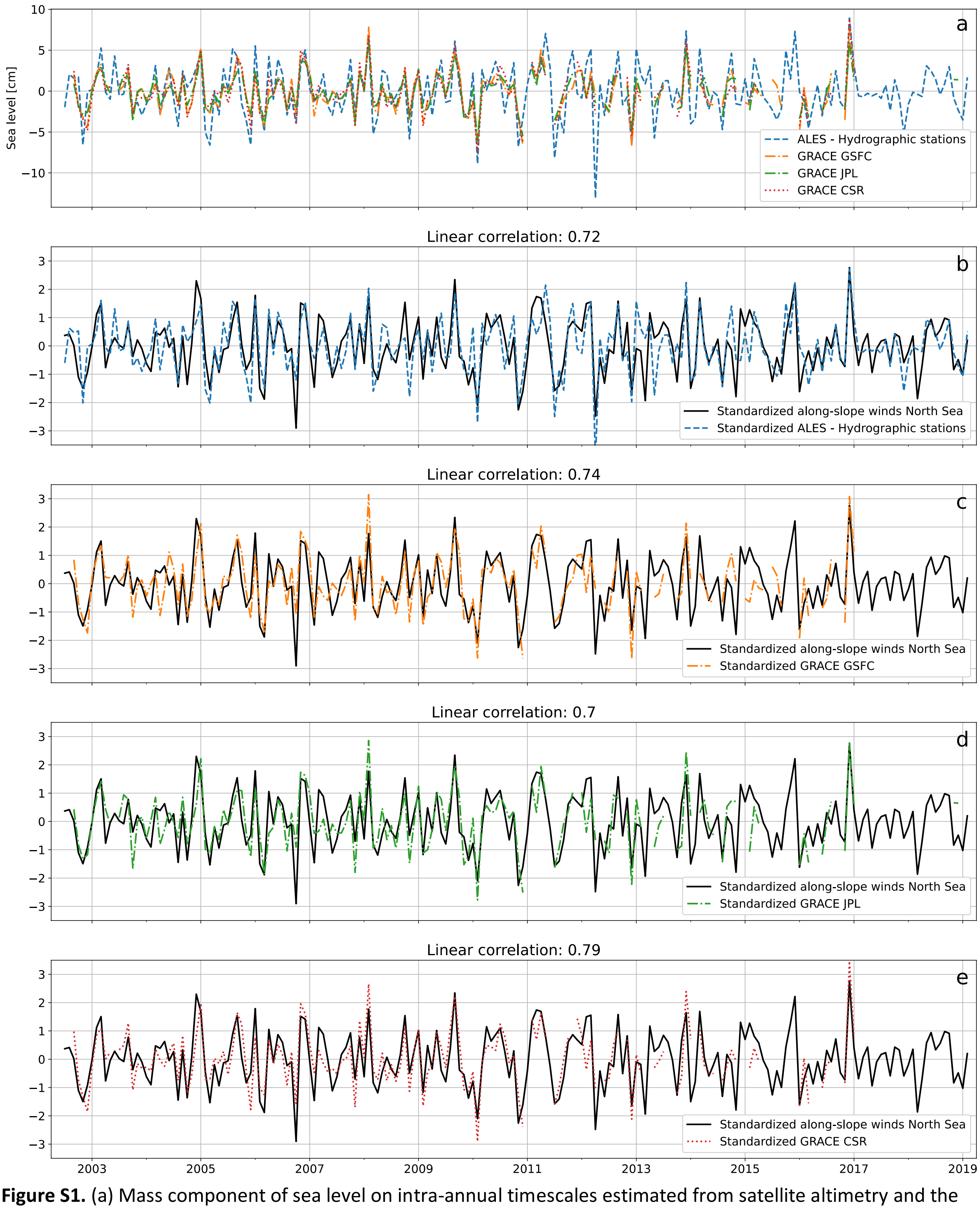

Figure S1. (a) Mass component of sea level on intra-annual timescales estimated from satellite altimetry and the hydrographic stations (blue, dashed line), and from the GSFC mascon solution (orange, dashed-dotted line), the JPL mascon solution (green, dashed-dotted line), and the CSR mascon solution (dotted, red line). (b) Standardized mass component of sea level on intra-annual timescales estimated from satellite altimetry and the hydrographic stations compared with the standardized along-slope component of the along-slope wind stress averaged along

the North Sea section of the shelf break (thick, black line). (c), (d), and (e) are like (b), but they show the mass component of sea level obtained from the GSFC's, the JPL's, and the CSR's mascon solution respectively.

B - Mass component of sea level: spatial variations

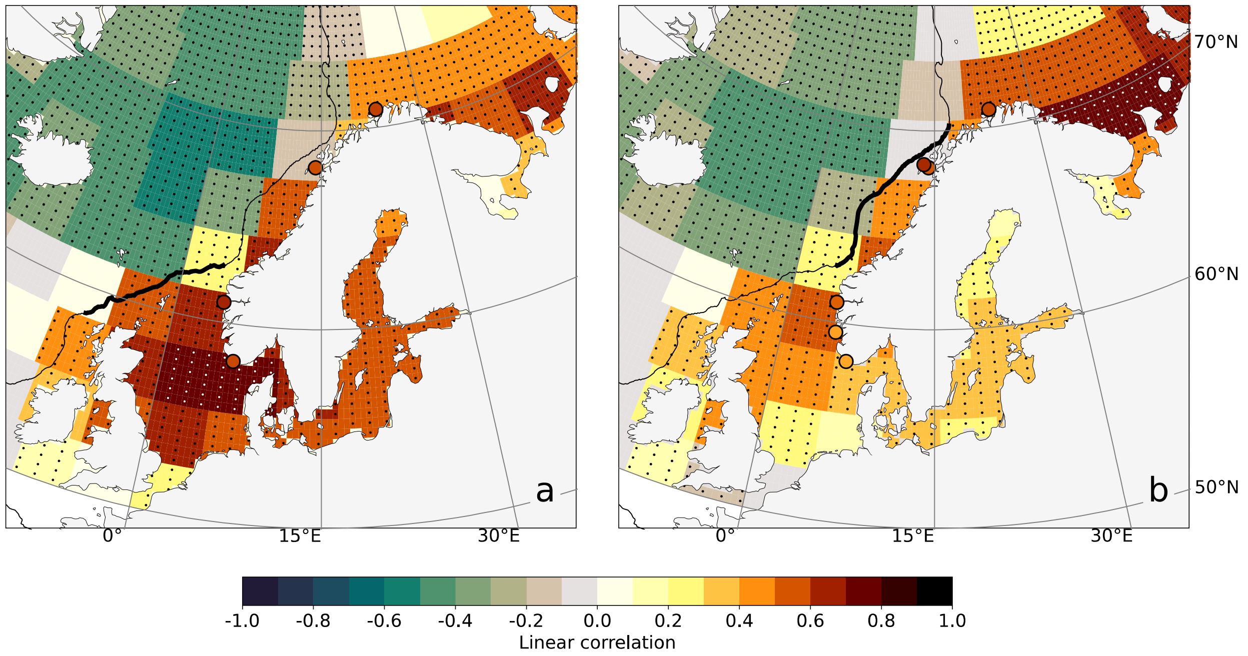

Figure S2. (a) Linear correlation coefficient between the along-slope component of the wind stress averaged along the North Sea section of the northern European continental slope (the black, thick line) and the mass component of sea level from JPL's mascon solution (shading) and from the combination of ALES and the hydrographic stations (circles) on intra-annual timescales. (b) Same as (a), but the linear correlation is performed with the along-slope wind stress averaged along the Norwegian section of the northern European continental slope. The black and white dots identify the regions where the linear correlation is significant at a 0.05 significance level. The black, thin line shows the location of the continental slope, depicted by the 500m isobath.

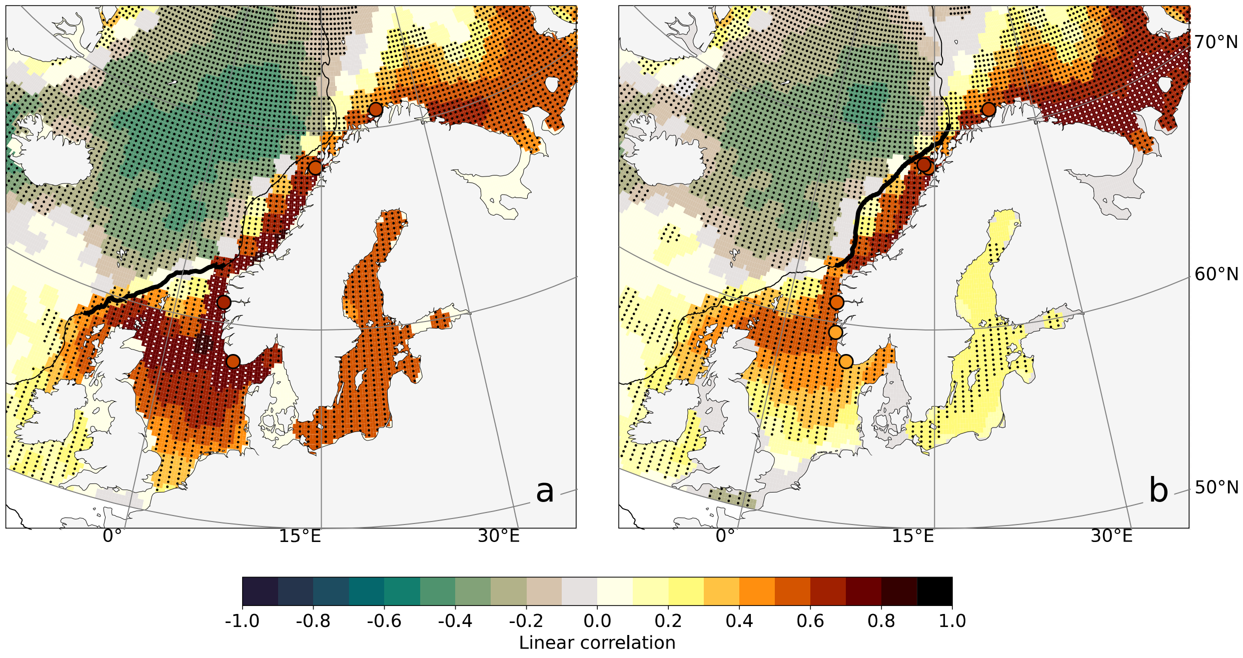

Figure S3. (a) Linear correlation coefficient between the along-slope component of the wind stress averaged along the North Sea section of the northern European continental slope (the black, thick line) and the mass component of sea level from CSR's mascon solution (shading) and from the combination of ALES and the hydrographic stations (circles) on intra-annual timescales. (b) Same as (a), but the linear correlation is performed with the along-slope wind stress averaged along the Norwegian section of the northern European continental slope. The black and white dots identify the regions where the linear correlation is significant at a 0.05 significance level. The black, thin line shows the location of the continental slope, depicted by the 500m isobath.

C) Comparison with NAO

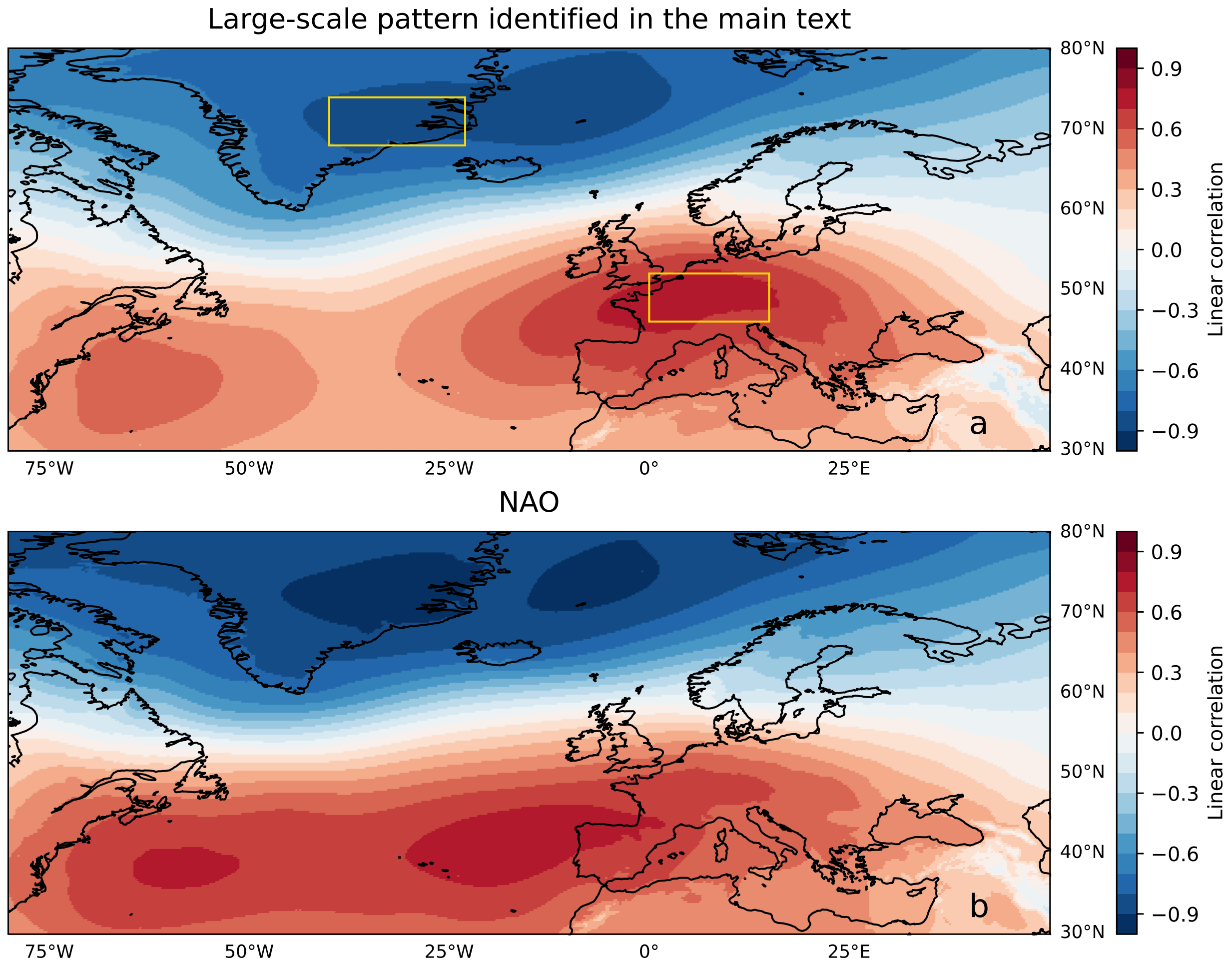

Figure S4. (a) Linear correlation coefficient between the gridded intra-annual monthly MSLP and the intra-annual along-slope component of the along-slope wind stress averaged over the North Sea section of the northern European continental slope. (b) First leading mode of intra-annual monthly MSLP over the region 80°W-50°E and 30°N-80°N expressed as correlation of the corresponding principal component with the input data set at each grid point.
